# The nerve cells in gastrointestinal cancers: from molecular mechanisms to clinical intervention

**DOI:** 10.1038/s41388-023-02909-x

**Published:** 2023-12-11

**Authors:** Yang Lyu, Fuda Xie, Bonan Chen, Wing Sum Shin, Wei Chen, Yulong He, Kam Tong Leung, Gary M. K. Tse, Jun Yu, Ka Fai To, Wei Kang

**Affiliations:** 1 Department of Anatomical and Cellular Pathology, State Key Laboratory of Translational Oncology, Sir Y.K. Pao Cancer Center, Prince of Wales Hospital, The Chinese University of Hong Kong, Hong Kong, China; 2 Institute of Digestive Disease, State Key Laboratory of Digestive Disease, Li Ka Shing Institute of Health Science, The Chinese University of Hong Kong, Hong Kong, China; 3 CUHK-Shenzhen Research Institute, Shenzhen, China; 4 Guangdong Provincial Key Laboratory of Digestive Cancer Research, Digestive Diseases Center, Scientific Research Center, The Seventh Affiliated Hospital of Sun Yat-sen University, Shenzhen, China; 5 Department of Pediatrics, The Chinese University of Hong Kong, Hong Kong, China; 6 Department of Medicine and Therapeutics, The Chinese University of Hong Kong, Hong Kong, China

**Keywords:** Gastrointestinal cancer, Cancer microenvironment

## Abstract

Gastrointestinal (GI) cancer is a formidable malignancy with significant morbidity and mortality rates. Recent studies have shed light on the complex interplay between the nervous system and the GI system, influencing various aspects of GI tumorigenesis, such as the malignance of cancer cells, the conformation of tumor microenvironment (TME), and the resistance to chemotherapies. The discussion in this review first focused on exploring the intricate details of the biological function of the nervous system in the development of the GI tract and the progression of tumors within it. Meanwhile, the cancer cell-originated feedback regulation on the nervous system is revealed to play a crucial role in the growth and development of nerve cells within tumor tissues. This interaction is vital for understanding the complex relationship between the nervous system and GI oncogenesis. Additionally, the study identified various components within the TME that possess a significant influence on the occurrence and progression of GI cancer, including microbiota, immune cells, and fibroblasts. Moreover, we highlighted the transformation relationship between non-neuronal cells and neuronal cells during GI cancer progression, inspiring the development of strategies for nervous system-guided anti-tumor drugs. By further elucidating the deep mechanism of various neuroregulatory signals and neuronal intervention, we underlined the potential of these targeted drugs translating into effective therapies for GI cancer treatment. In summary, this review provides an overview of the mechanisms of neuromodulation and explores potential therapeutic opportunities, providing insights into the understanding and management of GI cancers.

## Introduction

With the growing understanding of tumorigenesis, there has been an increasing focus on the role of the tumor microenvironment (TME) in this process. The TME consists of not only the tumor itself but also the intricate niche surrounding it. This niche includes nerve cells, surrounding blood vessels, immune cells, fibroblasts, signaling molecules, and the extracellular matrix (ECM) [1]. Recent research has shed light on the influence of not only the immune microenvironment but also the neural microenvironment on the development and occurrence of various solid tumors. Studies have indicated an upsurge in nerve density in these tumors [2], and it has been observed that tumors with higher innervation tend to exhibit increased rates of invasion and metastasis [3]. Although the overall significance and mechanisms of tumor-related neuroplasticity are not yet fully comprehended, these discoveries suggest that the nervous system actively contributes to tumor development, thereby offering new avenues for the treatment of tumor development through the modulation of nerve-cancer cross-talk.

Gastrointestinal (GI) cancers are a group of cancers characterized by a high incidence and affecting various sites within the GI tract. Common types of GI cancers include esophageal cancer (ESCC), gastric cancer (GC), colorectal cancer (CRC), liver cancer (LC), and pancreatic cancer (PDAC) [4]. Projections for the year 2023 in the United States show that GI cancers are expected to have the highest number of new cases and estimated deaths (348,840 new cases and 172,010 deaths). These figures account for 17.81% and 28.21% of the total number of cancer cases, respectively. Among the mentioned cancers, CRC has the highest mortality rate and is relatively easier to diagnose. According to data from the American Cancer Society, CRC ranks third among both male and female cancer patients in the United States [5]. PDAC has the second highest fatality rate among GI cancers, with a 5-year survival rate of only 12% between 2012 and 2018. LC and ESCA also have low survival rates, at only 21% [5]. In comparison, GC and CRC have relatively higher 5-year relative survival rates, at 33% and 65%, respectively. However, GC exhibits significant geographic disparities, primarily affecting low- and middle-income countries, which may correlate with living standards. Reports suggest that in China, GC was the leading cause of cancer deaths until 2004, when it was replaced by lung cancer (ranking as the third leading cause in 2016). Although the overall mortality rate of GC is declining in China, the death rate among GC patients in rural areas remains higher than in urban areas [6].

Research has shown that gut microbiota dysbiosis is associated with the development of various GI cancers. For example, Helicobacter pylori (*H. pylori*) is classified as a Group I carcinogen, and its infection is a major cause of GC [7]. An increased relative abundance of Desulfovibrio vulgaris has also been linked to promoting CRC [8]. Additionally, factors such as smoking, obesity, genetic predisposition, and poor dietary habits contribute to an increased risk of various GI cancers, including GC, LC, PDAC, and CRC [9, 10]. Despite significant advancements in cancer treatment in recent decades, challenges persist in the diagnosis, drug resistance, and recurrence of tumors. ESCC patients often have a poor prognosis, as they are frequently diagnosed with local invasion or lymph node metastasis [11].

In this review, our objective was to explore the connections between the nervous system and GI cancer. To start, we provided a comprehensive overview of the components that comprise the nervous system, offering insights into its neurogenic organizational structure. Subsequently, the intricate relationship between GI tumors and the nervous system was discussed from multiple perspectives. Valuable insights into potential therapeutic targets were obtained by elucidating the specific neural pathways that innervate the GI tract and their involvement in cancer development. Ultimately, we aspire for this review to advance the field, resulting in improved outcomes and a better quality of life for patients affected by GI cancer.

## The interplay between the nervous system and GI system

### Classifications of the nervous system

**Fig. 1:**
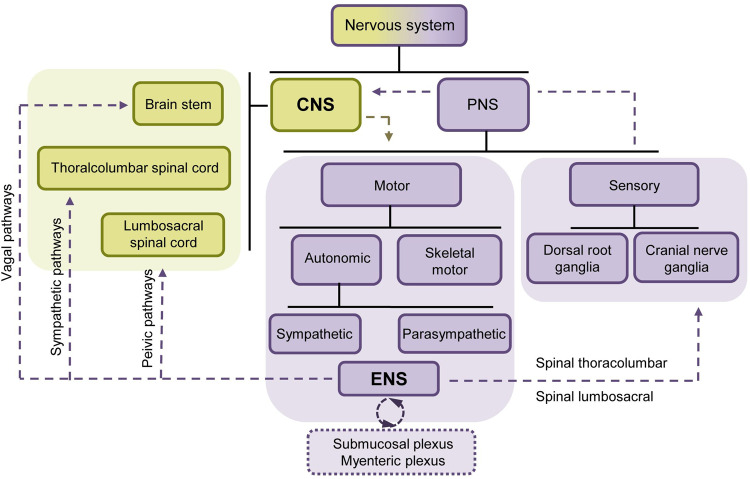
Relationship between the ENS and CNS. The diagram depicts the intricate interaction between the central nervous system (colored in yellow) and the peripheral nervous system (colored in purple), showcasing their extensive exchange of information. Both the vagus nerve and the enteric nervous system are composed of various neural components. While the enteric nervous system operates as a remarkably comprehensive neural network, it is important to note that it is not entirely autonomous. This neural network proves proficient in facilitating localized intestinal reflexes through intrinsic nerve ganglia or plexuses, notably in the small and large intestines, but not in the stomach and esophagus. Moreover, it serves as a conduit for relaying signals, employing pathways that involve the sympathetic ganglia, the vagus nerve, and other routes to establish connections with the central nervous system. Reciprocally, neural signals from the central nervous system are also conveyed through these nerves to the enteric nervous system.

In mammals, the nervous system primarily comprises the central nervous system (CNS) and the peripheral nervous system (PNS). The CNS, consisting of the brain and spinal cord, serves as the central information processing center, while neurons located outside the brain and spinal cord are classified as part of the PNS (Fig. 1) [12].

The brain functions as the central hub for processing information in the CNS, while the spinal cord plays a vital role in signal transmission and coordinating reflex actions. Situated within the spinal column, the spinal cord connects various organs and tissues through distinct segments. The upper thoracic spinal cord segments innervate both the head and thoracic regions, whereas the lower segments innervate the abdominal and pelvic regions [13].

The PNS is regarded as an extension of the CNS. For instance, the preganglionic neurons of the parasympathetic nervous system (PSNS) emerge from specific segments of the cranial and sacral nerves. Originating from the sacral spinal cord, the PSNS sends preganglionic fibers that extend to the pelvic viscera, controlling the function of the distal colon and the genitourinary system [14]. Within the PNS, the autonomic nervous system (ANS) is responsible for possessing self-regulatory capabilities and has a wide distribution, consisting of numerous pre and postganglionic regions. Different divisions of the ANS respond rapidly to local changes through specific neurotransmitters, aiding the CNS in regulating functions such as blood pressure, heart rate, vascular reactivity, and GI function.

### The enteric nervous system (ENS) functions predominantly in the digestive system

The enteric nervous system (ENS), which is unique from other internal organs, is an independent nervous system that has evolved within the GI tract to handle complex behaviors. It has been classified as one of the three major branches of ANS [15]. The ENS is capable of integrating neuronal activity and controlling various GI activities, such as intestinal peristalsis (movement of the intestines), mucosal fluid movement, immune response, and mucosal secretions, without depending on CNS [16].

The ENS is a highly intricate neural network comprising over 100 million neurons and over 400 million enteric glial cells (EGCs). These neurons and glial cells are interconnected in various ways, forming an independent system mainly concentrated in interconnected ganglia found beneath the mucosa and within the layers of the muscles [12]. In addition to assisting CNS in regulating GI functions, the ENS also allows the GI tract to possess a certain level of self-mediated reflex responses and self-regulation, thereby maintaining GI stability [12]. Often referred to as the “second brain” of humans, the ENS is capable of orchestrating the processes of intestinal tissue movement without requiring direct input from the brain or spinal cord [17]. Within the GI tract, each nerve fiber within the ENS consists of a diverse group of neurons with distinct neurochemical encoding and functions. Interstitial chains connect these nerve fibers with neighboring neurons, facilitating signal transmission within the submucosal and myenteric plexuses [18].

The submucosal plexus and myenteric plexus are responsible for regulating blood vessels and intestinal movements, respectively [16]. When the GI environment changes, the ENS transmits signals to target cells downstream through neural conduction and the release of neurotransmitters. This enables the ENS to control the motility, secretion, and sensory functions of the intestine, ultimately achieving local regulation of intestinal homeostasis. Additionally, intestinal epithelial cells also play a role in regulating the ENS. For example, DCLK1-positive tuft cells exhibit characteristics similar to neurons in terms of their gene expression. These cells are capable of secreting neurotransmitters, which can influence the function of the ENS [11].

### Communications between CNS and ENS in normal GI tract development

While the ENS is capable of functioning independently, it still maintains bidirectional communication with CNS. This communication is facilitated by neural connections between the ENS, CNS, and sympathetic ganglia. These connections can be broadly categorized as the vagus nerve, spinal thoracolumbar, and spinal lumbosacral pathways. Generally, local reflexes in the GI tract are transmitted to the CNS through the sympathetic nervous system and ENS. These systems provide feedback signals to GI cells by neurotransmitters (acetylcholine (ACh), nitric oxide, and serotonin) and neuropeptides (neuropeptide Y, substance P (SP), and vasoactive intestinal peptide).

The vagus nerve connects the brainstem to the GI tract, serving as a communication pathway between the brain and the gut. It transmits sensory information from receptors in the GI tract to CNS. The vagus nerve also plays a crucial role in regulating GI functions, including the control of appetite, GI contractions, and the secretion of acid, hormones, enzymes, and other substances. Interestingly, the bidirectional information exchange between the brain and the gut is unbalanced, with ~90% of the vagus nerve fibers being afferent, transmitting signals from the gut to the brain [19]. This indicates that the brain receives input from receptors along the GI tract.

Cholinergic signaling primarily regulates target cells within the GI tract through the vagus nerve. Notably, the release of ACh extends beyond nerve-related cells. As mentioned earlier, DCLK1^+^ tuft cells act as a non-neuronal source of ACh within the intestinal tract [20]. These tuft cells have an extended lifespan and possess unique stem cell-like abilities, allowing them to facilitate the repair of damaged intestinal epithelial cells [21]. Additionally, the cytokines produced by these cells enhance intestinal contractility and influence inflammatory responses in the GI mucosa [11, 22]. These studies underscore the crucial importance of cross-regulation between the epithelial cell and neuronal system in maintaining intestinal homeostasis.

The CNS and the ENS exhibit both similarities and differences. As integral nervous system components, they share the capacity to recognize and respond to a wide range of neurotransmitters, including ACh, adrenaline, and catecholamines. However, the CNS and ENS are situated in different anatomical locations and serve distinct functions, while collectively contributing to the overall homeostasis and coordination of the body through bidirectional communication.

## Regulating functions of CNS in GI cancer progression

Generally speaking, the mutual regulation between CNS and GI cancers primarily occurs through the influence on immune responses, endocrine functions, vascular formation, or disruption of nervous system functionality. Several decades ago, it was observed that certain behaviors in organisms, such as stress, chronic depression, and lack of social support, can influence the development and progression of tumors [23]. Survey reports showed that individuals with an optimistic mindset had a 16% lower risk of cancer-related mortality than pessimistic individuals. In recent years, researchers have made progress in explaining the impact of mental stress on tumor progression beyond initial clinical phenotype observations. In GI cancers, increased work-related stress and insufficient sleep have been associated with a higher incidence of CRC and ESCC [24].

**Fig. 2:**
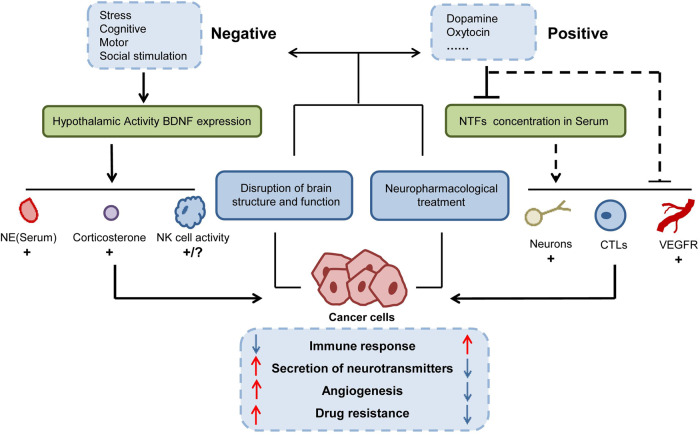
The interplay between the CNS and GI tumors. The CNS exercises regulatory influence over downstream cellular and molecular transformations linked to GI tumors, achieved through the modulation of pertinent neurotransmitter secretion. This regulatory grasp extends to encompass factors like nerve density, vitality of immune cells, and the activation of factors associated with angiogenesis. CNS resonates GI tumorigenesis through various aspects, including the modulation of drug resistance within GI tumors, the intensity of immune responses, and the potential for vascular growth. Meanwhile, the tumors themselves possess the capability to shape the configuration of the CNS reciprocally.

Current research on molecular mechanisms suggests that stress can disrupt multiple physiological axes, including the brain-adipocyte brain-derived neurotrophic factor (BDNF) /leptin axis, the sympathetic-adrenal axis, and the hypothalamic-pituitary-adrenal axis. This disruption would lead to a reduction in cellular immunity, thereby promoting the development of CRC, PDAC, and tumor resistance [25]. Conversely, positive emotions were found capable of lowering the concentration of neurotrophic factors in the bloodstream, increasing the proportion of CD8^+^ cytotoxic T lymphocytes, and inducing microglia/macrophage activation, thereby inhibiting the progression of GI cancers [26]. The intricate relationship between the CNS and GI tumors is briefly illustrated in Fig. 2.

In addition to stress stimuli, various emotional stimuli can influence the development of GI cancers through the hypothalamus. Oxytocin, a neuropeptide secreted by oxytocin neurons in the hypothalamus, plays a role in regulating anxiety and depression [27]. In addition to stress stimuli, various emotional stimuli can influence the development of GI cancers through the hypothalamus [28]. These findings suggest that chemical stimulation of oxytocin neurons, such as with cephalotin, may serve as a promising therapeutic approach for treating CRC [28]. Dopamine (DA), a catecholamine (also known as the stress neurohormone) and a precursor to epinephrine, is secreted by the CNS. Dopamine has been shown to inhibit tumor angiogenesis and impede the invasion and migration of GC by suppressing VEGFR-2 phosphorylation and the EGFR/AKT/MMP-13 signaling pathway [29]. Dopamine receptors are a class of G protein-coupled receptors with five subtypes (DRD1-DRD5), and are associated with the growth and prognosis of a variety of GI cancers. High expression of DRD2 is associated with poor prognosis in CRC patients. Depletion of DRD2 in CRC can down-regulate β-catenin/ZEB1 signaling and inhibit tumor cell growth [30]. In addition, several neurotransmitters widely present in the CNS have been detected in GI tumors.

## Various patterns of the PNS-mediated regulation on GI tumorigenesis

The abnormal phenotypic alterations of PNS have been universally observed in GI cancers at the histological level. It has been found that as cancer progresses from precancerous lesions to overt cancer, there is a significant increase not only in blood vessel density but also in nerve density. Subsequently, the relationship between nerve density and cancer invasiveness has been confirmed in various tumors, including prostate, colon and rectal, head and neck, breast, pancreatic, gastric, and lung cancer. This suggests that the PNS may play a crucial role in tumorigenesis.

**Fig. 3:**
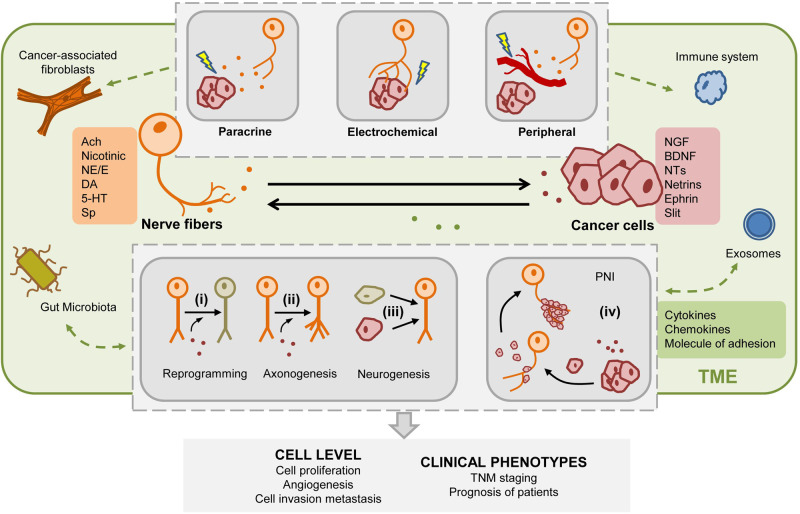
Interplay between tumors and neural components. The regulation between nerve cells and tumors operates in both directions, forming a bidirectional relationship that is significantly influenced by the tumor microenvironment. Transformations in nerve cells induced by tumor-secreted factors encompassing (i) Conversion of nerve cell types; (ii) Hypertrophy of neurons or axonal elongation; (iii) Transformation of non-neuronal cells into nerve cells; (iv) Infiltration, envelopment, and penetration of tumor cells into nerves. The nerve cell-derived regulation on cancer cells is divided into three modes: paracrine-dependent regulation, electrochemical signals, and peripheral circulatory factor-dependent regulation. The interplay between neural and tumor components involves various cells and molecules, including those from the immune system, gut microbiota, and exosomes. These multifaceted contributors collectively engage in the interplay process, ultimately giving rise to a spectrum of biological phenomena.

The PNS can be functionally divided into the sensory (afferent) nervous system and the motor (efferent) nervous system. As our understanding of the relationship between PNS and tumors advances, it has been found that tumor progression can be influenced by both electrochemical signaling and the secretion of first messengers like neurotransmitters or n eurotrophins factors. Enabled by these regulatory functions, PNS is identified as a crucial participator in GI TME remodeling and GI cancer progression. The interaction between neural components and the TME of GI cancers is depicted in Fig. 3.

### Sensory nervous system-mediated sensory innervation

The cell bodies of primary sensory neurons are located in the cranial and spinal ganglia, and their primary function is to transmit information from target organs to the CNS through nerve fibers [14]. Among various types of sensory neurons, visceral sensory nerve fibers play a significant role in regulating GI cancers. Their cell bodies are primarily located in the tuberosity and jugular ganglia and are mainly carried by the vagus nerve. By detecting changes in homeostasis and conveying sensory information to the brainstem, the visceral sensory nervous system controls essential bodily functions, such as circulation, temperature regulation, digestion, respiration, and immune responses [31]. As for GI cancer progression, existing studies mainly focus on their associations with capsaicin and neuropeptide SP.

Capsaicin, a compound found in chili peppers, selectively activates sensory neurons that are sensitive to it. The response of these neurons varies with the concentration of capsaicin: it stimulates unmyelinated axons at low concentrations and desensitizes unmyelinated fibers at higher concentrations [14, 32]. The precise mechanisms of regulating tumors are not yet fully understood. However, several studies have suggested that capsaicin treatment or vagus nerve-mediated denervation can lead to the inactivation of unmyelinated C fibers, thereby increasing the risk of PDAC, GC, CRC, and bile duct cancer (CHOL).

SP, released by sensory neurons, plays a role in pain perception. Its receptor, the neurokinin-1 receptor, is widely expressed on immune cells, neurons, and tumor cell surfaces [33]. Studies have revealed that sensory axons can be recruited by pancreatic intraepithelial neoplasia (PanIN) lesions. Through SP/neurokinin-1 receptor signaling and the JAK-STAT pathway, this recruitment can activate and promote the proliferation of neuroendocrine PanIN cells, thereby facilitating the progression of PanIN lesions in PDAC.

### ENS innervation: mediated by various first messengers

The interaction between ENS and GI cancer is intricate and diverse. Neurotransmitters or related substances secreted by neurons or EGCs can regulate the progression of tumors [34]. Correspondingly, those or other neural–related substances from GI cancer or TME can also affect neural reprogramming, new neural recruitment, and axonogenesis. This process is facilitated by a range of neurotrophic factors, axon guidance molecules, as well as neurotransmitters. Table 1 shows the target cells express specific receptors for various neural factors, activating downstream signaling pathways.

**Table 1:** Axon guidance molecules, neurotropic factors, and neurotransmitters in the development of GI cancers.

Molecules		Receptors	Cancer Types	Function	Signaling pathways	References
Axon guidance molecules						
Netrins	Netrins-1	DCC and UNC5B/C	GC, CRC, HCC, PDAC	Metastasis, Proliferation, Migration and Invasion	YAP pathway, ERK/MAPK, PI3K/AKT pathway and FAK activation	[36, 37, 40, 42]
			PDAC			
	Netrins-4 (β-netrin)	Neogenin (Neo)	CRC, GC	Angiogenesis and Proliferation	JAK/STAT, PI3K/AK, ERK/MAPK pathway	[44, 45]
Eph/Ephrin	EphrinA-1	EphA (A1, A2, A3, A4, A5)	CRC, GC	Proliferation, EMT, Migration, and Invasion	STAT3/VEGF pathway	[51, 56]
	Ephrin-B	EphB (B2, B4, B6)	CRC, GC	Migration and Invasion		[53, 54]
Semaphorins	Sema5A/6B/3E/4C/6D/4D/3C	plexins	PDAC, GC, CRC	Proliferation, Tumor differentiation, Invasion, Migration, Angiogenesis and Apoptosis	PI3K/Akt/uPA pathway	[57–59]
	Sema3B/3A/4G					
Slit - Roundabout (Robo)	Slit1/2/3	Robo1	T-P:HCC, PDAC, CRC, ESCC	EMT, Invasion, Metastasis and Angiogenesis	Wnt/β-catenin, PI3K/Akt, FAK NEK9 activation, RAFTK/Pyk2 and Src-mediated E-cadherin pathway	[64, 66–68]
		Robo2	HCC, PDAC	T-cell infiltration	TGF-β signaling pathway	[63, 72]
		Robo3 / Rig-1	PDAC	Metastasis	Wnt/β-catenin pathway	[69]
Neurotrophins						
Brain-derived neurotrophic factor (BDNF)		TrkB, p75NTR	ESCC, GC, CRC, PDAC	Proliferation, EMT	BDNF/TrkB pathway	[77, 81]
Nerve growth factor (NGF)		TrkA, p75NTR	PDAC, HCC, ESCC, GC,	Proliferation, Metastasis	Ach/NGF/M3R/YAP axis and RET/RAS/MAPK pathway	[78, 79, 85, 94]
Neurotropic factors (NTs)	Neurotrophin-3 (NT-3)	TrkC, p75NTR	CRC, PDAC	EMT	MMP1/NT-3/TrkC pathway	[82, 86]
GDNF family of ligands (GFL)	Glial cell line-derived neurotrophic factor (GDNF)	Rearranged during Transfection (RET) in complex with glial cell line-derived (GDNF) family receptors α (GFRα)	PDAC	Perineural spread, perineural invasion	RET/RAS/MAPK pathway	[34]
	Neurturin (NRTN)		CRC			[74]
Neurotransmitter						
Glutamate		mGluRs	COAD, PDAC	Invasion and Migration	KRAS/MAPK pathway	[87, 89]
		iGluRs	GC, COAD	Cell cycle		[88]
GABA		Metabotropic receptor (GABA-B)	HCC	Cell cycle	cAMP/p21, MAPK/AKT pathway	[92]
		Ionotropic receptors (GABA-A or GABA-C)	PDAC, GC	Proliferation and invasion		[90, 91]
Dopamine		G-protein-coupled dopamine receptors (DRs)	COAD GC	Proliferation	EGFR/AKT/MMP-3 and β-catenin/ZEB1 pathway	[29]

#### Axon guidance molecules

Axon guidance molecules, such as Netrins, Ephrins, Semaphorins, and Slit, have been demonstrated to play a role in the tumorigenesis and development of various GI cancers.

Starting with Netrins, as secreted proteins and membrane-bound proteins, Netrins not only influence neural cell differentiation but also impact tumor progression and stages. Notably, in late-stage GC and most of CRC, defective Netrin-1-dependent receptors DCC and UNC5C result from abnormal gene methylation [35, 36]. Netrin-1 can stimulate YAP signaling, the ERK/MAPK signaling cascades, and the PI3K/AKT pathway, thereby promoting the proliferation and invasion of GC cells [37–39]. Hypoxia-induced Netrin-1 activation can also trigger NF-κB and p65 downstream of AKT, promoting epithelial-mesenchymal transition (EMT) in hepatocellular carcinoma (HCC) cells [40]. Furthermore, Netrin-1 can facilitate CRC liver metastasis through the miR-329-3p/Netrin-1-CD146 complex [41]. However, the role of Netrin-1 is intricate and varies across tumorigenesis stages in PDAC. Research reveals low expression in stages I and II PDAC, increasing in stages III and IV. Netrin-1 exhibits both pro- and anti-tumor functions in PDAC [42, 43]. In comparison to Netrin-1, Netrin-4 has garnered less attention in GI cancer research. Nonetheless, some studies suggest that Netrin-4 elicits a similar effect on GC as Netrin-1, activating JAK/STAT, PI3K/Akt, and ERK/MAPK pathways to promote GC cell proliferation [44]. In contrast to Netrin-1, Netrin-4 serves as a tumor suppressor in CRC by inhibiting angiogenesis, hinders primary tumor growth, liver and lung metastases [45, 46].

The Eph-ephrin signal pathway involves Erythropoietin-producing hepatoma (Eph) receptors and their ligands, ephrin [47]. In summary, the majority of members in the Eph-ephrin family function as oncogenes in GI cancers. For instance, upregulation of EphA4, Ephrin-B2, EphA2, and EphrinA-1 is indicative of a poor prognosis in GC and contributes to the migration and invasion of CRC cell lines [48–52]. The EphB/ephrin-B combination serves as a valuable marker for dysplastic/oncogenic transformation in GC [53]. On the molecular level, EphA3 can activate the STAT3/VEGF signaling pathway, while EphA2 mediates CAFs-induced gastric tumorigenesis. The tumor suppressor gene function of Eph-ephrin family members is primarily evident in CRC. In CRC,EphB6, EphA5, and EphA1 are positively correlated with survival time [54, 55]. And EphA1 downregulation enhances the proliferation of HRT18 human rectal adenocarcinoma cells [56].

As for Semaphorins, they are classified into eight classes (classes 1-7 and V-viral), and various subtypes of Semaphorins exhibit abnormal expression patterns in GI tumors. For instance, Sema5A is overexpressed in GC and PDAC [57, 58], whereas Sema4G is down-regulated in CRC [59]. Sema4D is overexpressed in both GC and CRC [60, 61]. Ke Wang et al. comprehensively summarized the roles of Semaphorins in GC: Sema5A/6B/3E/4C/6D/4D/3C act as tumor promoters, facilitating tumor progression by promoting GC invasion, metastasis, or angiogenesis. In contrast, Sema3B/3A functions as GC tumor suppressor [62].

Within the Slit-Robo signaling framework, Slits (Slit1-3) interact with roundabout receptors (Robo1-4) in GI cancers. Among these, Robo1 has been extensively investigated and established as a cancer promoter in various malignancies. In precancerous intestinal lesions and during tumor progression, Slit2/Robo1 signaling triggers the Wnt/β-catenin pathway by down-regulating E-cadherin and induces EMT [61]. In HCC, ESCC, PDAC and GC, the Robo1 signaling axis activation can promotes cancer progression, and metastasis [63–67]. Conversely, deubiquitinating and stabilizing ROBO1 in CRC can inhibit tumor migration [68]. In PDAC, high expression levels of Robo1 and Robo3 are associated with reduced patient survival. It is worth noting that there exists a negative correlation between Robo1 and Robo3 expression, suggesting a potential contradiction within the Slit/Robo pathway [69]. In contrast to Robo1 and Robo3, Robo2 primarily functions as a tumor suppressor. High Robo2 expression often correlates with longer survival in PDAC and HCC patients [70]. In PDAC, Robo2 inhibits the activation of multiple downstream signaling pathways, such as MAPK and PI3K, by disrupting the interaction between TGF-β and HGF-MET, thereby impeding tumor growth [71, 72]. Overall, the Slit-Robo signaling pathway plays a crucial role in GI cancer, with its specific functions being subtype-dependent.

#### Neurotrophins

Neurotrophins are a class of secreted proteins that play essential roles in the nervous system’s survival, growth, development, and plasticity. Four Neurotrophins: nerve growth factor (NGF), BDNF, NT3, and GDNF, were listed in Table 1. These Neurotrophins exert their effects by binding to their receptor Trk, which belongs to the growth factor receptor (GFR) superfamily and possesses tyrosine kinase activity. In PDAC, CRC, the expression of these neurotrophic factors, including NGF and GFL neurotrophic factors, is upregulated [73, 74]. They can induce the spread of cancer cells around nerves by stimulating the GFRα3/RET receptor or promote neural invasion through activation of the RET-Ras-MAPK pathway, thereby facilitating PDAC metastasis and correlating with a poor prognosis [75, 76]. In ESCC and GC, TrkB is overexpressed and plays a role in tumor growth, metastasis, and EMT via the BDNF/TrkB pathway [77]. In ESCC, the high secretion of NGF is associated with increased expression and activation of TrkA, as well as decreased expression of p75NTR [78, 79]. Neurotrophins play a regulatory role in CRC, primarily through TrkB and TrkC receptors. BDNF/TrkB and BDNF/sortilin co-localize on the plasma membrane of CRC cell, activating downstream AKT signaling pathways and promoting CRC cell proliferation and survival [80, 81]. TrkC is frequently methylated in various CRC cell lines and functions as a conditional tumor suppressor in CRC [82]. There is limited research on the influence of neurotrophins in HCC; however, experimental studies have shown that TrkA acts as an oncogene in HCC, while p75NTR has potential tumor-suppressive functions [83–85]. And in PDAC, inhibiting the NCT-3/TrkC signaling pathway can suppress tumor development [86].

#### Neurotransmitters

Neurotransmitters are released by peripheral and autonomic nerves, which can be classified into three groups according to their chemical structure: (1) amino acids (Such as ACh, glutamate, and γ-aminobutyric acid (GABA)); (2) biogenic amines (Such as dopamine, norepinephrine, epinephrine, and serotonin) (3) neuropeptide (Such as SP, neuropeptide Y, and vasoactive intestinal polypeptide). Here, we focus on the roles of glutamate and GABA in GI cancers.

These two types of neurotransmitters, widely present in CNS and corresponding receptors, have been detected in various tumors. Among them, GABA is the major inhibitory neurotransmitter, while glutamate is the most important excitatory neurotransmitter in CNS. Glutamate is highly expressed in PDAC tissues and promotes tumor migration by activating the KRAS-MAPK signaling pathway [87]. Ionotropic glutamate receptors (iGluRs) show differential expression in CRC and GC, whereas high expression of mGluR4 in CRC leads to resistance to 5-Fluorouracil [88, 89].

There are three different receptors (A, B, and C) of GABA. Generally, GABA-A promotes tumor proliferation, while GABA-B inhibits tumor growth. In GC and PDAC, GABA activates the MAP kinase pathway through GABA-A receptors to promote cell proliferation [90, 91]. In vitro and in vivo experiments have shown that the use of GABA-B agonists can inhibit tumor development in LC [92].

### Other ANS-mediated autonomic nerve innervations

#### Parasympathetic innervation: cholinergic signaling

The PSNS exerts dominant regulation over the GI tract, primarily through the vagus nerve-mediated cholinergic signaling. Nevertheless, the receptors involved in neuro-tumor regulation in response to cholinergic signaling may vary depending on the tumor type. The receptors that receive ACh signals include muscarinic ACh receptors (mAChRs) and nicotinic ACh receptors (nAChRs), with mAChRs being more prevalent in regulating GI cancers. Within the mAChR family, GC samples exhibit high expression of CHRM1 and CHRM3. In CRC, CHRM3 activation is observed, while CHRM1/2/3 is associated with poor prognosis in LC [93].

Recent advancements have shed light on the molecular mechanisms of cholinergic signaling in GI cancer progression. Gastric tumors can release NGF, which fosters nerve recruitment and the proliferation of Dclk1 cells, resulting in increased ACh release [94]. ACh, acting via the CHRM3 receptor, modulates crucial regulatory factors in tumor growth, such as the Wnt and Notch signaling pathways, or utilizes AMPK signaling, thereby subsequently promoting tumor cell proliferation. These findings suggest the potential therapeutic use of anti-cholinergic drugs for GC and related malignancies.

**Fig. 4:**
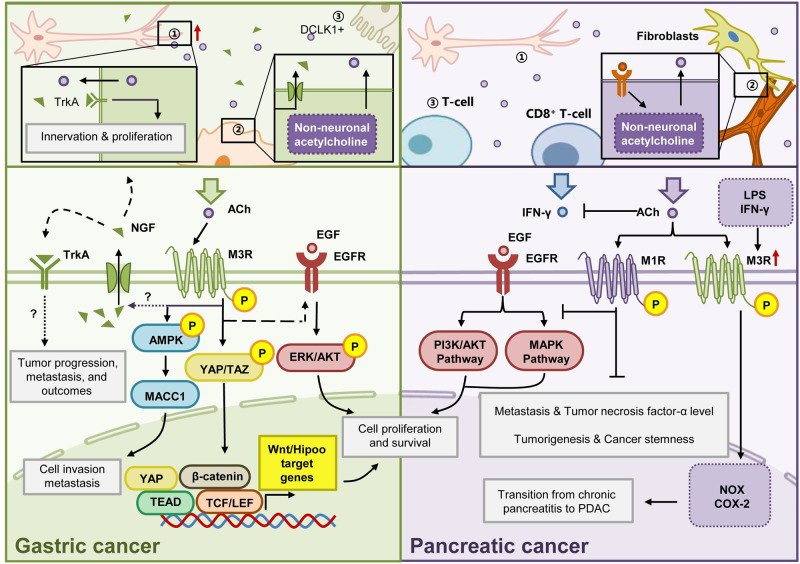
Cholinergic signaling in gastric and pancreatic cancer. In GC progression, ACh is secreted by three distinct sources: neurons, cancer cell-derived choline acetyltransferase, and DCLK1-positive tuft cells. ACh present in the local environment has the capacity to bind to the type 3 muscarinic receptors (M3Rs) situated on the surface of tumor cells. This binding event triggers a cascade of downstream signaling pathways, culminating in the stimulation of NGF production and subsequent release. This NGF-mediated process plays a pivotal role in fostering the proliferation of nerve cells, thereby contributing to the amplification of acetylcholine content within the immediate local environment and completing the positive feedback cycle. TrkA is also present on the surface of GC cells, which is also activated by NGF and influences a variety of phenotypes associated with cell proliferation and metastasis. In PDAC, ACh is also sourced from three distinct origins: neurons, fibroblasts, and T cells. Following release, ACh binds to the type 1 muscarinic receptors (M1Rs) in cancer cells. Notably, this binding event impedes the activation of downstream PI3K/AKT and MAPK pathways that EGFR typically activates. In addition, M3R was highly expressed in PDAC, and LPS and IFN-γ could induce this expression. When M3R is activated, it can induce the expression of COX-2 and NOX in cells, among which COX-2 can induce the formation of chronic pancreatitis and carcinogenesis.

The effects of the PSNS on GI cancer vary considerably and are contingent upon the particular type of tumor. Studies have indicated that in PDAC, the regulatory role of the PSNS contradicts that in GC and LC. Notably, after vagus nerve resection in PDAC patients, a shortened survival time was observed. The CHRM1 receptor, which is closely associated with the prognosis of PDAC, transmits cholinergic signals to inhibit the EGFR/MAPK and PI3K/AKT signaling pathways, thereby extending patient survival (Fig. 4) [95]. Therefore, cholinergic agonists are being considered as potential adjunctive treatments for PDAC. However, studies have demonstrated that activation of ACh receptors, α3, α5, α7 – nAChRs receptors induces PDAC cell proliferation, indicating sophisticated patterns may be involved in PSNS regulation.

#### Sympathetic innervation: epinephrine signaling

In most GI cancers, the signaling output of the sympathetic nervous system is mediated through the secretion of neurotransmitters like epinephrine/norepinephrine or isoprenaline, which have an impact on tumor initiation and progression. Through binding to β2-adrenergic receptors (β2-AR), epinephrine/norepinephrine can activate multiple downstream signaling pathways and exert an influence on the progression of GI cancers.

In GC patients, norepinephrine (NE) induces gastric EMT and enhances the invasive and migratory capabilities of GC cells through the activation of multiple pathways, including β2-AR-HIF-1α-Snail signaling, β2-AR-STAT3-CD44 signaling, and β2-AR-MMP7 signaling [96–98]. Similarly, activation of the β2-AR by another agonist isoproterenol triggers the initiation of EMT and the expression of associated markers, accelerating the malignancy of GC cells and ultimately tumor progression [97].

Upregulation of NE is also demonstrated in PDAC. Remarkably, PDAC cells demonstrate the capacity to synthesize NE, leading to its localized accumulation and subsequent activation of signaling pathways involved in cell proliferation, growth factor release, and apoptosis inhibition [99, 100]. Meanwhile, increased adrenaline activity can promote the progression of pancreatic diseases from the PanIN stage to adenocarcinoma [101].

## The feedback regulation of tumor cells on nerves

### Perineural invasion

Peripheral invasion (PNI) is present in various types of GI cancers. According to former reports, the incidence of PNI could reach 98%, 75%, and 33% in PDAC, CHOL, and CRC, respectively [102, 103]. It is characterized by the infiltration and accumulation of tumor cells around peripheral nerves, which is facilitated by the secretion of neurotransmitters and neuropeptides by nerve terminals [104]. PNI plays diverse roles in the progression of GI cancers and is associated with high invasiveness and various clinical features, including the 5-year survival rate, tumor size, and lymph node metastasis. It has been identified as an independent adverse prognostic factor in GC and CRC [105, 106].

Mechanically, the occurrence of PNI is associated with the expression of various cytokines, chemokines, and adhesion molecules. Hepatocyte growth factor (HGF), the ligand of c-Met, has been implicated in promoting PNI in PDAC. Recent studies illustrated the promotion pattern of the HGF/c-Met signaling pathway in PDAC: the overexpression of c-Met leads to activation of the mTOR/NGF axis, resulting in nerve recruitment and enhanced invasion of cancer cells into nerves [107, 108]. Meanwhile, chemokines like CCL21 and CXCL10 facilitate early PNI formation by promoting cancer cell migration towards sensory neurons. The expression of their respective receptors (CCR7 and CXCR3) can also influence patients’ perception of pain [109]. In GC, co-expression of CXCL8 and MMP9 often implies a poor prognosis and serves as a detection marker for PNI [110]. Moreover, in vitro co-culture experiments have revealed that vascular cell adhesion molecule-1 (VCAM1) contributes to PNI and accelerates GC progression by promoting interactions between nerve cells and tumor cells [111]. Furthermore, the connection between extracellular vesicles and PNI has been observed. For instance, extracellular vesicle-derived miR-128-3p has been demonstrated to induce the activation of TGF-β/SMAD and JAK/STAT3 signaling pathways in CRC, thereby promoting perineural infiltration and lymphovascular invasion [112].

### Neoneurogenesis, axonogenesis and neural reprogramming

Three predominant biological processes are involved in cancer cell-originated neuron regulation: neoneurogenesis, axonogenesis, and neural reprogramming. Neoneurogenesis, also known as innervation, refers to the formation of new functional neurons promoted by tumor cells through the release of neurotrophic factors. The exact origin of these new neurons is still under debate. Axonogenesis involves inducing axonal growth in surrounding nerve cells by tumor cells. Neural reprogramming, on the other hand, entails converting non-neuronal cells into neurons.

In previous studies, it was mentioned that the removal of PNS, sensory nerves, or vagus nerves, as well as the blockade of related receptors, can impact the progression of GI cancer. However, subsequent studies have shown that this effect cannot be solely attributed to the removal of pre-existing neurons [113]. Further research revealed that nerves appearing in tumors may originate from the CNS or mesenchymal stem cells (MSCs) [114, 115], and the neural density in solid tumors often correlates with prognosis (higher neural density indicating improved survival rates in CRC) [116]. These findings suggest the potential significance of neural reprogramming, axonogenesis, and neoneurogenesis in neuro-tumor regulation. Under appropriate TME conditions, tumor cells recruit suitable cells like MSCs to differentiate into neurons or promote axonal extension and growth [117]. While the molecular mechanisms underlying axonogenesis and neural development are not yet fully understood, it is known that stimulation of acetylcholine-nerve growth factor can induce PNI and increase neural density in GC and PDAC [73]. Recent experimental studies in mice have indicated that tumor cell-released extracellular vesicles play a role in regulating axonogenesis and neurogenesis. Specifically, several studies have shown that tumor cell-released extracellular vesicles can initiate the process of axonogenesis. Furthermore, vesicles containing EphrinB1 protein have been found to enhance the activity of axonogenesis [118].

## The cross-talk between the nervous system and TME in GI tumorigenesis

**Fig. 5:**
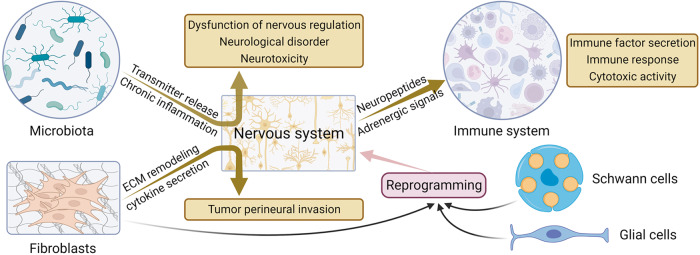
The cross-talk between the nervous system and TME. The indirect regulation of nerve-tumor interactions is summarized through the TME, acting as the link between nerves and tumors, influencing the development of GI cancer. TME comprises nerve cells, immune cells, microbial populations, and cancer-associated fibroblasts. Dysbiosis of the gut microbiota can influence psychoneurotoxicity and provoke immune responses. Within the TME, fibroblasts, glial cells, and Schwann cells can undergo reprogramming to transform into nerve cells, contributing to nerve repair, regeneration, or peripheral invasion to a certain extent. While immune cells have the potential to transform into neural cells, in most cases, the immune system is influenced by neural signals, leading to alterations in its immune response to tumors.

The intricate interplay between the TME and tumor cells results in various effects encompassing various aspects of tumor development and progression. The latest study by Bin He et al. showed that cancer cells can acquire stemness properties by exploiting neural signals in the microenvironment and pointed out that this process is facilitated through the cAMP-responsive element pathway [119]. The indirect modulation of interactions between the nervous system and tumors can be summarized as the TME acting as a bridge, exerting influence on the development of GI cancer. The TME comprises diverse components, including nerve cells, immune cells, microbiota, and tumor-associated fibroblasts. These components can interact and mutually influence one another, thereby directly or indirectly impacting the development of GI cancer (Fig. 5).

### Gut microbiota

Emerging research suggests that the interaction between the nervous system and tumors offers new insights and potential therapeutic strategies for cancer treatment. Dysregulation of the gut microbiota has been implicated in the development of various cancers. For instance, *H. pylori* has been identified as a significant factor in developing GC and PDAC. It triggers the accumulation of inflammatory factors in the stomach, leading to abnormal gene methylation in cancers [7]. It may also cause mutations in the KRAS gene and activate tumor-promoting genes (STAT3, c-myc, Bcl-xL) in the pancreas [120]. CRC has been associated with an increased abundance of sulfate-reducing bacteria caused by a high-fat diet, while LC has shown connections with imbalances in Escherichia coli in the GI tract [8, 121].

The Gut Microbiota-brain Axis, which represents the communication between the gut microbiota and the brain, has garnered recognition in recent years. Research has demonstrated that the gut microbiota can interact with the brain through several mechanisms, including neurotransmitter production, immune regulation, inflammation response, and blood-brain barrier permeability [122, 123]. This bidirectional communication has significant implications for human health and the development of neurological disorders, including anxiety disorders, depression, autism spectrum disorders, Parkinson’s disease, and Alzheimer’s disease [123–126]. Furthermore, imbalances in the gut microbiota have been implicated in emotions, cognitive function, and behavior, resulting in symptoms such as anxiety, depression, and impaired cognitive function [125, 127, 128].

The Gut Microbiota-brain Axis and the interplay between the gut microbiota and tumor regulation imply the potential for indirectly regulating GI cancer development through cross-talk between the nervous system and gut microbiota. Research findings indicate a link between the gut microbiota and psychoneurotoxicity associated with cancer treatment. The gut microbiota can influence the development of psychoneurotoxicity and its associated symptoms through various mechanisms. Dysbiosis of the gut microbiota can disrupt the normal function of the intestinal mucosal barrier, enabling harmful bacteria and metabolites to enter the circulatory system, thereby impacting neurotransmitter production and function [129–131]. Additionally, dysbiosis of the gut microbiota can trigger chronic inflammatory responses that can further influence the function of the nervous system and the balance of neurotransmitters [130–132].

It is crucial to note that while there exists a link between the gut microbiota, neural regulation, and tumor progression, the specific mechanisms and treatment strategies demand further research and validation. Additional clinical trials and human studies are necessary to assess the safety and efficacy of utilizing gut microbiota in cancer treatment.

### Immune cells

The intestine is widely acknowledged as the largest immune organ. As mentioned earlier, the immune system is involved in diverse regulatory processes, including neuro-tumor regulation. In recent years, there has been growing evidence suggesting that disrupted neuro-immune interactions contribute to the development of cancer and metastasis. Notable examples of this are the neuropeptides SP and calcitonin gene-related peptide (CGRP). These neuropeptides demonstrate tumor characteristics and play a significant role in regulating the immune system in sensory neurons.

For example, research studies indicate that high levels of SP have been shown to facilitate the migration of natural killer cells (NK cells), stimulate the secretion of immune-related factors such as IFN-γ and IL-12, and boost the cytotoxic activity of immune cells, including cytotoxic cells and NK cells [133, 134]. SP can activate cell-mediated cytotoxic immunity against pathogens and immune responses against precancerous cells during the immune response to pathogenic agents [135]. In summary, the release of SP by sensory neurons can bolster the cytotoxic immune response against advanced cancer, thereby modulating tumor development.

Moreover, the interaction between neuro-tumor-immune processes extends beyond the aforementioned examples. ACh can also be synthesized by T cells, specifically those that express β2-AR. Upon stimulation by adrenergic neural signals, these T cells secrete ACh, which subsequently plays a role in the immune regulation of tumors. For instance, ACh has the ability to facilitate tumor cell proliferation and inhibit the expression of tumor necrosis factor in macrophages [136, 137].

### Fibroblasts

In solid tumors, the tumor stroma constitutes more than 50% of the tumor composition. Alongside endothelial cells, immune cells, and ECM, cancer-associated fibroblasts (CAFs) play a significant role. CAFs, a distinct cell type within the TME, can either promote or suppress tumor characteristics through ECM remodeling or cytokine secretion. The functions of CAFs encompass various roles, including stimulating angiogenesis, controlling immune suppression, regulating cancer cell metabolism, and promoting or inhibiting cancer cell proliferation and metastasis [138].

In GC, CAFs serve as a source of IL-17, which activates the JAK2/STAT3 signaling pathway and thereby enhances the invasion and migration of GC cells [139]. In a study conducted by Shen et al., YAP1 expression level was upregulated when co-culturing CAFs and PDAC cells. Furthermore, this study confirmed that CAFs can enhance perineural invasion in PDAC by activating the YAP1/TEAD1/NGF pathway [140]. CAFs also exert an influence on CRC. Although the maturation of these cells does not significantly correlate with the prognosis of CRC patients, they have the ability to impact the invasion pattern of the cancer [141]. In PDAC, there is an upregulation of CCL26 expression in CAFs. This upregulation activates the PI3K/AKT/mTOR pathway, consequently fostering perineural invasion and facilitating lymph node metastasis, particularly in advanced tumor stages [142].

Several studies have attempted to develop new cancer treatment strategies based on the interaction between CAFs and TME. Interestingly, efforts to deplete CAFs have resulted in unexpected outcomes in the advancement of PDAC. Instead of inhibiting tumor growth as anticipated, CAF depletion has led to the promotion of immunosuppression and, consequently, a decrease in overall survival [143]. Recent studies have identified the presence of CAFs with contrasting functions in PDAC. Notably, depleting fibroblast activation protein (FAP)^+^ CAFs has been found to reduce the survival rates of PDAC patients. Conversely, the depletion of α-smooth muscle actin (α-SMA)^+^ CAFs has shown promise in improving the survival rates of individuals with PDAC [144]. These findings emphasize the importance of precisely targeting specific subtypes of CAFs and highlight the complex nature of CAF involvement in cancer therapy.

### Non-neurocyte-originated neurocyte transformation

The impact of various tissue and cell components within the TME on regulating neural tumors extends beyond indirect regulation via molecule release. Some specialized cells, such as macrophages, T cells, fibroblasts, and glial cells, can also actively engage in generating new nerves through direct reprogramming. This direct reprogramming also referred to as transdifferentiation, has been extensively investigated, particularly for transforming fibroblasts into neurons. Studies have demonstrated that specific transcription factors (OCT4, SOX2, c-MYC, p53) [145, 146], RNA molecules (such as miR-9 and miR-124) [147], and recombinant proteins [148] can induce the direct reprogramming of fibroblasts into neurons or dopamine neurons. Most of these processes are related to molecular mechanisms such as the JAK/STAT signaling pathway and the Wnt signaling pathway. However, there is still insufficient research on the transformation of CAFs into functional neurons in the TME. Previous studies have shown that glial cells can be reprogrammed and converted into neuronal-like cells (for example, astrocytes can differentiate into functional neurons). In neuro-tumor regulation, much research has been done on Schwann cells (SCs). SCs mainly participate in neuro-tumor regulation in two ways. One way is through involvement in PNI. One way is through involvement in PNI. As the main source of various cell movement and adhesion-related proteins, SCs promote the migration of SCs to cancer cells and release chemical molecules to facilitate tumor proliferation in PDAC with low expression of SLIT2. They also enhance the interaction between cancer cells and nerves, ultimately promoting the PNI process [149]. Another way is through reprogramming to participate in nerve repair and regeneration. When the peripheral nervous system is damaged, some SCs differentiate into repair SCs (rSCs) and guide the process of axonal growth and nerve regeneration [150]. In 2022, Philip Tang et al. revealed a process called macrophage-to-neuronal cell transformation. The study showed that under the induction of tumor secretions, a specific subset of tumor-associated macrophages that express Tubb3 and lose macrophage markers is generated in the TME. This process is dependent on Smad3 and is associated with cancer pain [151].

### Neuroendocrine tumors (NETs) in the gastrointestinal system

Neuroendocrine cells regulate body growth, development, and internal homeostasis by secreting hormones and peptides. In the GI system, over 14 types of neuroendocrine cells have been identified, and mutations in these cells contribute to the development of NETs [152]. The specific gene mutations promoting NET development remain unclear. Studies indicate that elevated expression of somatostatin receptors (SSTRs) and angiogenesis-related genes, including vascular endothelial growth factor (VEGF) and fibroblast growth factor, is characteristic of NETs [153]. Higher tumor differentiation correlates with increased gene expression. Additionally, the pathogenesis of NETs varies across different sites. For instance, hereditary small intestinal neuroendocrine neoplasms (NENs) result from IPMK inactivation, whereas strong PROX1 expression in rectal NENs is likely to promote tumor progression [154, 155]. Clinically, most NENs (e.g., those with MEN1 gene mutations) grow slowly, while some (e.g., those with VHL gene mutations) have malignant potential [156, 157]. Overall, NENs exhibit high heterogeneity.

## Therapeutic opportunities

A comprehensive understanding of neuro-tumor regulation provides valuable insights and potential avenues for cancer treatment. The complex interaction between the nervous system and tumors is widely recognized as a crucial factor in tumor progression and treatment response. Investigating neuroregulatory mechanisms can unveil novel targets and pathways, forming the basis for developing innovative treatment strategies.

### Tumor resistance to neurological intervention

Chemotherapy is a primary therapeutic approach for treating GI cancers. However, the effectiveness of chemotherapy can vary among individuals, and the development of drug resistance poses a significant clinical challenge in cancer treatment. Mouse experiments have shown that the activation of adrenaline can influence the sensitivity of tumor cells to chemotherapy by modulating apoptotic pathways. In the case of breast cancer (BC), the relationship between the β2-AR and resistance to the anti-HER2 monoclonal antibody trastuzumab has been elucidated. Upregulation of β2-AR by HER2 can activate the PI3K/AKT/mTOR pathway, leading to tumor resistance to trastuzumab, a first-line treatment for both GC and BC [158]. Subsequent experiments combining β-blockers with trastuzumab have improved BC patients’ survival rates [159]. These findings highlight the significance of adrenergic signaling in modulating chemotherapy response and drug resistance in cancer. Understanding the underlying mechanisms and identifying therapeutic strategies to overcome drug resistance are crucial research areas for enhancing chemotherapy’s effectiveness in GI cancers.

Moreover, ACh inhibitors have been studied to improve the sensitivity of GC to chemotherapy [160]. Both clinical and mouse experiments have shown that administering botulinum toxin, an inhibitor of the ACh pathway, can effectively suppress GC recurrence, enhance patient survival, and optimize chemotherapy outcomes [3]. These findings suggest a potential association between neuroregulation and tumor drug resistance, indicating that targeting neuro-related pathways could enhance tumor sensitivity to drugs. Further investigation into adrenergic and cholinergic signaling pathways may lead to novel therapeutic strategies, ultimately improving treatment outcomes and patient survival rates in GI cancers.

### New neuromodulation-targeted therapy

Therapeutic approaches targeting neurotrophic factor receptors have emerged due to the increasing understanding of their role in neuro-tumor regulation. Ongoing clinical investigations mainly focus on NGF and its associated receptor TRKA. Larotrectinib and entrectinib, two small molecule inhibitors of Trk receptors, have been suggested as effective treatment options [161]. Ongoing research focuses on using these inhibitors to block NGF-TrkA or BDNF-TrkB signaling pathways, thus inhibiting tumor development [94]. These drugs have already received FDA approval for treating Trk fusion-positive tumors [161].

Furthermore, neuroregulation therapy shows promise in the management of cancer-related pain. Tumor-related pain is a prevalent challenge for cancer patients, and neuroregulation techniques such as spinal cord stimulation, dorsal root ganglion stimulation, and peripheral nerve stimulation provide non-pharmacological and non-surgical alternatives for pain relief [162]. Several studies suggest that these neuroregulation techniques can relieve cancer-related pain and improve patients’ quality of life [163].

In conclusion, a deep understanding of the interaction between nerves and tumors has provided new insights and methodologies for cancer treatment. This progress has guided the development of clinical interventions, transitioning from the historically problematic denervation surgeries of the 19th century to modern adjunctive therapies that target tumor innervation. This transformation has not only increased the effectiveness of cancer treatment but has also provided new therapeutic options for clinical implementation. Additionally, the use of neuromodulation techniques for pain management adds another dimension. These techniques have the potential to alleviate moderate to severe cancer-related pain and improve the quality of life for individuals undergoing cancer treatment. With the deepening research in this field, neuromodulation is positioned to provide patients with more effective treatment options, thereby improving the overall prognosis for individuals with cancer.

## Conclusions and perspectives

In summary, the regulation of GI cancers by the nervous system is primarily mediated through ANS. Neuro-related cells, mediators, and components of the TME actively participate in the complex interplay between the nervous system and digestive tract tumors. This bidirectional communication and influence highlight how tumors can exploit neural pathways to their advantage in the pursuit of growth and progression. While this field of research has garnered significant attention and provided valuable clinical guidance, several unresolved issues persist.

Further research is necessary to deepen our understanding of the effectiveness and underlying mechanisms of gut neuroregulation. Despite some progress, the treatment outcomes for gut diseases such as inflammatory bowel disease, obesity, nausea, and gastroparesis remain inconsistent [164]. Numerous factors, including individual differences, disease types, and disease severity, may impact the efficacy of treatments for these complex conditions. Exploring novel treatment modalities and identifying personalized approaches will be crucial for improving therapeutic outcomes. Additionally, careful consideration must be given to the safety and long-term effects of neuroregulation techniques. While certain neuroregulation techniques, like gastric electrical stimulation for upper GI disorders, have been utilized, understanding the potential risks and evaluating the long-term effects is paramount [165]. Ensuring both the safety and effectiveness of these treatments poses a significant challenge in advancing neuroregulation techniques for clinical application.

Moving forward, future studies should prioritize enhancing our understanding of neuroregulation in the TME. This would encompass the exploration of innovative treatment strategies and the fostering of interdisciplinary collaborations. By doing so, we can optimize treatment outcomes and substantially enhance the quality of life for patients suffering from GI cancer. It is through these endeavors that we can hope to unlock the full potential of neuroregulation techniques and improve patient care in this challenging disease landscape.
